# Aseptic meningitis with recurrent headache episodes, vomiting, and central fever as first manifestation of isolated neurosarcoidosis: a case report

**DOI:** 10.1186/s12883-024-03794-x

**Published:** 2024-08-28

**Authors:** Athina-Maria Aloizou, Theresa Anne Gabriel, Carsten Lukas, Ralf Gold, Jeremias Motte

**Affiliations:** 1 Neurology Department, St. Josef Hospital Bochum, Ruhr University Bochum, Gudrunstr. 56, 44791 Bochum, Germany; 2 Institute of Neuroradiology, St. Josef-Hospital Bochum, Ruhr University Bochum, Bochum, Germany

**Keywords:** Aseptic meningitis, Neurosarcoidosis, Sarcoidosis, Neuroimmunology

## Abstract

**Background:**

Neurosarcoidosis is a rare entity, usually within the context of systematic sarcoidosis. Isolated neurosarcoidosis and especially a manifestation with pachymeningitis is a notable rarity.

**Case Report:**

A 26-year-old patient presented to the emergency department with acute onset, recurrent episodes of occipital headaches spreading over the whole cranium and vomiting without food consumption, for three days. The clinical examination did not reveal any neurological deficits. The laboratory exams showed no pathological findings. A CT examination with angiography did not detect any acute intracranial or vessel pathology. A lumbar puncture was performed to rule out subarachnoid hemorrhage. The results showed a lymphocytic pleocytosis of 400/µL, elevated protein levels of 1077 mg/dL and reduced glucose levels (CSF: 55 mg/dL, Serum: 118 mg/dL). Extensive infectiological examinations did not reveal any signs of infection, including Borrelia spp. and M. tuberculosis. No positive auto-antibodies or vasculitis-related auto-antibodies were detected. The CSF analysis showed negative oligoclonal bands but an isolated increase in β2-microglobulin, neopterin, and IL-2R levels. The MRI examination revealed a dural gadolinium-enhancement, pronounced in the basal cerebral structures and the upper segment of the cervical spine, consistent with neurosarcoidosis. Corticosteroid treatment rapidly led to a significant improvement of the symptoms. No systemic manifestations of sarcoidosis were found.

**Conclusions:**

This case report aims to highlight aseptic meningitis with atypical, acute onset headache attacks as a possible manifestation of isolated neurosarcoidosis. Neurosarcoidosis is a clinical entity that requires prompt treatment to avoid permanent neurological deficits.

## Introduction/background

Sarcoidosis is a multisystemic granulomatous disease that commonly affects the lungs, skin, and eyes, with an estimated incidence of 10–20/100,000 [1]. Neurosarcoidosis occurs in 5–20% of patients with systemic sarcoidosis. However, the available information on neurosarcoidosis is limited, as the available single-center studies carry a significant degree of variability [2]. The cranial nerves are most commonly affected in neurosarcoidosis, though it can also manifest as polyneuropathy, hydrocephalus, seizures, meningitis, and myelitis. An isolated neurosarcoidosis, without systemic manifestation, represents a notable rarity [3–5]. Diagnosing isolated neurosarcoidosis can be challenging, due to atypical manifestations, numerous mimics, and the inherent difficulty of acquiring tissue samples for biopsy. Diagnostic criteria were recently made available and require a rigorous exclusion of other possible causes [4]. In this case report, we describe a young patient with acute-onset, recurrent headaches, vomiting, and central fever, without meningism signs. The patient’s CSF and MRI findings were compatible with aseptic meningitis and neurosarcoidosis. The purpose of this report is to raise awareness on this atypical clinical manifestation of isolated neurosarcoidosis.

## Case presentation

A 26-year-old male patient with no prior medical history, including migraine or other headache disorders, presented to our emergency department with recurrent headache attacks for the past three days. The first attack had an acute onset with no identifiable triggering factors, and an intensity of 7–9/10. The headache was initially located in the occipital region and then spread throughout the entirety of the cranium. Phono- or photophobia were not reported. The patient then presented recurrent headache episodes of this character, in the morning and in the evening, while medication such as ibuprofen could only slightly improve the pain. Additionally, the patient reported daily vomiting episodes, occurring even without food consumption. No other symptoms, namely fever, diarrhea, or neurological deficits, were reported. Nicotin, alcohol, and drug consumption were denied. The patient reported working as a security guard in a refugee camp.

**Fig. 1:**
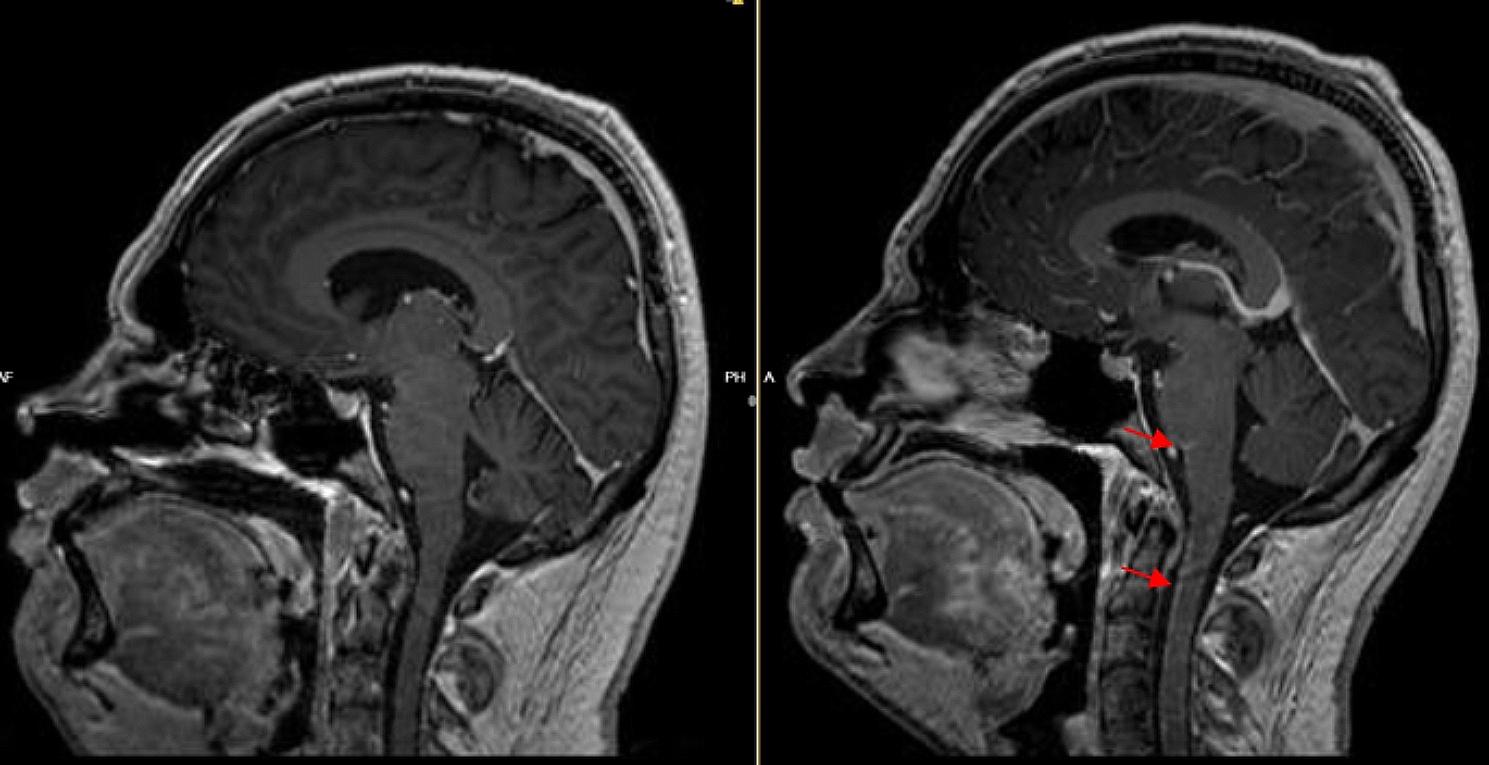
T1-weighted MRI sequences with Gadolinium enhancement. The left image demonstrates the follow-up MRI after approximately 8 weeks, with improvement of the meningeal enhancement (red arrows), pronounced at the basal brain structures and the spinal cord

Physical examination showed no focal neurological deficits or meningism. Vital parameters were normal, and the initial laboratory examination revealed no pathological findings. A cerebral CT scan (computed tomography) with angiography was carried out, to exclude a cerebral hemorrhage, vascular pathology, and sinus venous thrombosis; no pathological findings were found. A lumbar puncture to exclude a subarachnoid hemorrhage with greater certainty was then performed. The CSF (cerebrospinal fluid) pressure was measured at 15mmHg. The initial CSF findings revealed a lymphocytic pleocytosis (400/µL) with elevated protein (1077 mg/dL) and reduced glucose levels (CSF: 55 mg/dL, Serum: 118 mg/dL), without erythrocytes or siderophages. Empirical treatment with acyclovir, ampicillin, and ceftriaxone was initiated. The PCR (polymerase chain reaction) examination of the CSF did not detect any common CNS (central nervous system) infectious agents, while additional specific examinations, including single-PCR for Herpes Simplex 1/2 and Varicella Zoster Viruses, Borrelia ASI, next generation sequencing, 16 S-rDNA (bacterial universal) PCR, and panfungal PCR, were also negative. The CSF culture was sterile, and a native MRI of the brain showed no pathological findings. The antimicrobial treatment did not result in any clinical improvement. Instead, the patient started experiencing a fever of up to 39 °C. Repeated blood and urine cultures were sterile, while a lung X-ray and abdomen CT were unremarkable. Due to the patient’s professional background, a Quantiferon test was performed, along with tests for West Nile Virus, Dengue Fever and Yellow Fever antibodies, all of which were negative. Syphilis, HIV, and Hepatitis B/C examinations were also negative. Two Doppler ultrasound examinations, also during an acute headache attack, did not detect any vasospasm. The lumbar puncture was repeated three days later, revealing comparable findings with the first puncture. The cytological examination did not reveal any malignant cells. Furthermore, the oligoclonal bands and an array of auto-antibodies regarding systemic autoimmune disorders, vasculitis, and autoimmune encephalitis were negative (antinuclear-ANA and extranuclear-ENA antigen antibodies, anti-MOG, -AQP4, -Hu, -Ri, -ANNA-3, -Tr/DNER, -Ma1, -Ma2/Ta, -GAD65, -Ampiphysin, -NMDA, -AMPA, -GABA-b-Receptor, -LGI1, -CASPR2, -IgLON5, -DPPX, -Myelin, -CARPVIII, -Glycin, -mGluR1, -mGluR5, -GABA-a-Receptor, -Rho GTPase activating protein 26, -Recoverin, -GluRD2, -Flotillin 1/2, -ZIC4, -ITPR1, -Homer3, -Neurochondrin, -Neurexin-3-alpha, -ERC1, Sez6l2, -AP3B2, -Contactin1, -Neurofascin 155/186, -ATP1A3, -KCNA2, -Dopamin Receptor 2 antibodies). A second MRI examination of the brain and cervical spine with gadolinium enhancement revealed dural gadolinium enhancement, pronounced in the basal cerebral structures and the upper segment of the cervical spine, with no evidence of other intracranial or intramedullary pathology. Notably, we observed an isolated increase of β2-microglobulin, neopterin, and IL-2R levels in CSF, with normal serum and CSF ACE (angiotensin converting enzyme) levels. Intravenous corticosteroid treatment with methylprednisolone was initiated due to suspicion of neurosarcoidosis, resulting in immediate symptom improvement reported by the patient. An affection of the peripheral nervous system and the lungs could be excluded by the means of electroneurography and bronchoalveolar lavage. The patient was discharged with oral prednisolone (1 mg/kg). The patient remained asymptomatic after six weeks but suffered from various corticosteroid adverse effects, namely weight gain, acne, peripheral edema, and facial swelling, so a corticosteroid tapering plan and infliximab were initiated. The decision to initiate infliximab was based on the patient’s young age and possible need for long-term therapy, keeping in mind potential long-term side-effects of other immunosuppressive medications, and his wish to father children in the near future. The patient tolerated infliximab well. A new MRI after approximately 8 weeks revealed an improvement of the dural gadolinium enhancement (Fig. 1). In the last follow-up, 8 months after diagnosis, the patient had suffered no relapses, and only complained of light, non-persistent headaches, while exhibiting no focal neurological signs. The oral corticosteroid therapy could be minimized, the infliximab treatment was carried on.

## Discussion

In this case, we discuss an atypical manifestation of isolated neurosarcoidosis, presenting with recurrent acute onset headaches, vomiting, and central fever, which were associated with an aseptic meningitis responsive to corticosteroids. Neurosarcoidosis usually presents in patients with systemic sarcoidosis, in approximately 5% of cases, making isolated neurosarcoidosis a rarity [6], while meningitis also appears in only approximately 16% of neurosarcoidosis cases [2]. Pachymeningitis is also rarer, with fever only seldom mentioned [7]. As such, the rarity of this patient lies in the atypical clinical manifestation, including recurrent acute headaches, central fever, and the absence of cranial nerve involvement, in the context of pachymeningitis in isolated neurosarcoidosis.

According to the widely used criteria by Zajicek et al. (1999) [8], the patient was diagnosed with “possible neurosarcoidosis”. In these criteria, a definite diagnosis can be set only on the basis of a biopsy, while a probable neurosarcoidosis is considered when evidence of systemic sarcoidosis is present, either through positive histology, including Kveim test, and/or at least two indirect indicators from Gallium scan, chest imaging and serum ACE. A rigorous exclusion of other possible causes and evidence of CNS inflammation is also required. However, it has been frequently intonated that even the pathological identification of granulomas is not 100% definite for the diagnosis of the disease [4]. Sensitive and specific biomarkers are lacking; ACE is the most commonly known test for sarcoidosis, with epitheloid and giant cells of the granulomas producing an abundant amount of the enzyme, respective of the granulomas present, and therefore often applied in disease monitoring [9]. However, its sensitivity and specificity in both serum and CSF are questionable, since several granulomatous diseases can lead to its increase [10], and the numbers of patients with elevated ACE have ranged from 30 to 80% among different studies [9]. As a 2016 meta-analysis reported, CSF ACE was increased in less than half of neurosarcoidosis cases, and serum ACE in about a third [2], with our patient also demonstrating normal values. Other aseptic meningitis cases with neurosarcoidosis also demonstrated normal ACE values as well [11], especially in serum [12, 13]. IL-2R could represent another useful marker, since the accumulation of activated T-cells and the subsequent stimulated expression of IL-2R is a long-known pathological characteristic of sarcoidosis [14]. Although not specific for sarcoidosis, it has shown potential as a diagnostic and prognostic marker in this disease [10]. Similarly, β2-microglobulin, as a marker of lymphocytic activation, has also been reported as elevated in sarcoidosis patients, with often normal values of ACE [15], while elevated neopterin, a product of monocyte activation, has also been noted in some sarcoidosis cases, albeit lacking specificity [16]. Neopterin is also individually expressed in the CNS, with no correlations between serum and CSF levels; for neuroinflammatory disorders, higher CSF levels are noted in both infections and autoimmune disorders, albeit more pronounced of infections [17]. This is consistent with the diagnosis of isolated neurosarcoidosis in our patient, where neopterin was only elevated in CSF.

Building on previous efforts to define criteria and commenting on the many faces of neurosarcoidosis with absence of definite radiological and laboratory markers, Stein et al. (2018) proposed an updated version of the criteria [4]. These also included peripheral nerve pathology and a more “vague” description of typical inflammatory findings, considering how some older tests are now obsolete in modern practice, while also requiring pathological confirmation of systemic sarcoidosis for “probable”, and a nervous system biopsy positive for sarcoidosis for a “definite” diagnosis. Applying these criteria, our patient still received a “possible neurosarcoidosis” diagnosis.

Neurosarcoidosis requires prompt treatment in order to avoid residual neurological deficits. In several case-reports of neurosarcoidosis with atypical manifestation, corticosteroid/immunosuppressive treatment led to full remission, with patients free of symptoms in the follow-up [18], though other patients only achieved partial remission [11] or developed chronic issues such as hypopituitarism, cognitive impairment, and paraplegia [5, 12, 19], highlighting the need of high clinical suspicion and early treatment initiation; notably, the 2016 meta-analysis reported a complete remission in only 27% of included neurosarcoidosis cases [2]. There is no universal consensus and no available randomized trials regarding long-term immunosuppression in neurosarcoidosis, which still remains an individualized decision based on clinical severity and patient profiles, with the goal of sparing corticosteroids and avoiding permanent disability. Azathioprine and methotrexate are “traditional” immunosuppressive agents commonly used, though their clinical effect can take months to appear, and anti-TNF (tumor necrosis factor) monoclonal antibodies are increasingly being introduced early in the immunosuppressive treatment of neurosarcoidosis [20]. Infliximab in particular has shown very good rates of clinical remission in regards to CNS involvement, also in refractory cases [21], and has in fact been the most commonly administered treatment in neurosarcoidosis, as revealed in a recent meta-analysis [22]. Due to the young age of our patient and his desire to have children in the near future, the gravidity of his pachymeningitis, and the side-effects of oral corticosteroids occurring early, a long-term and fast acting immunosuppression was deemed crucial, thus leading us in choosing infliximab.

## Conclusions

Though a notable rarity, isolated neurosarcoidosis should also be considered in cases of aseptic meningitis non responsive to antibiotic and antiviral treatment; ACE should not be considered as an absolute marker of sarcoidosis, with other markers such as IL-2R, β2-microglobulin, and neopterin in CSF being helpful in these cases.

## Data Availability

All data generated or analysed during this study are included in this published article.

## Abbreviations

ACE: angiotensin converting enzyme
CSF: cerebrospinal fluid
CT: computed tomography
IL-2R: interleucin 2 receptor, MRI: magnetic resonance tomography imaging
PCR: polymerase chain reaction
